# Contraceptive choices and satisfaction: a cross-sectional analysis of sociodemographic influences

**DOI:** 10.48101/ujms.v130.12656

**Published:** 2025-07-29

**Authors:** Maria Åkesson, Jan Brynhildsen, Tanja Tydén, Niklas Envall, Helena Backman, Yvonne Skogsdal

**Affiliations:** a Faculty of Medicine and Health, University Health Care Research Centre, Örebro University, Örebro, Sweden; b School of Medical Science, Örebro University, Örebro, Sweden; c Department of Obstetrics and Gynecology, Faculty of Medicine and Health, Örebro University, Örebro, Sweden; d Department of Women’s and Children’s Health, Uppsala University, Uppsala, Sweden; e School of Health and Welfare, Dalarna University, Falun, Sweden; f Department of Women’s and Children’s Health, Karolinska Institutet, Stockholm, Sweden; g Department of Clinical Sciences, Danderyd Hospital, Karolinska Institutet, Stockholm, Sweden; h Maternal Health Care Unit, Faculty of Medicine and Health, Örebro University, Örebro, Sweden

**Keywords:** Contraception, birth control, long-acting reversible contraceptives, patient satisfaction, sociodemographic factors

## Abstract

**Introduction:**

Investigating factors associated with contraceptive satisfaction is important to create a basis for tailored contraceptive counseling. In this study, we aimed to explore how sociodemographic characteristics affected women’s level of satisfaction and choice of different contraceptive methods, using data collected during a randomized controlled trial (RCT) in the region Örebro County, Sweden.

**Methods:**

This cross-sectional study utilized data from a previously conducted RCT. Eligible participants were women aged 20–40 years who sought contraceptive counseling. All women who participated in the RCT and completed a follow-up questionnaire were included in the analysis.

**Results:**

Between February 2015 and March 2016, 1,946, participants were enrolled in the trial, with 1,198 (61.6%) completing the 2-month follow-up questionnaire. Overall, 81.3% of women reported being ‘very satisfied’ or ‘satisfied’ with their contraceptive method. Participants aged 27–40 years used long-acting reversible contraception (LARC) to a higher extent compared with those aged 20–26 years (adjusted odds ratio [aOR] 1.92, 95% confidence interval [CI] 1.44–2.56). Older age was associated with lower satisfaction (aOR 0.55, 95% CI 0.33–0.94).

Participants with a body mass index (BMI) ≥ 25 more often used LARC (aOR 1.68, 95% CI 1.24–2.28) but were also more likely to report no use of contraceptives at all (aOR 1.56, 95% CI 1.01–2.43) compared with BMI < 25. The level of satisfaction tended to decrease with increasing BMI. Country of birth and educational level were not associated with satisfaction.

**Conclusions:**

The use of LARC was more common among women with BMI ≥ 25 and older women. While BMI, education, and place of birth did not affect satisfaction, women aged 27–40 reported lower satisfaction. These findings contrast with prior studies and highlight the complex sociodemographic influences on contraception experiences.

## Introduction

Women’s satisfaction with their contraceptive method is a crucial factor for continuation and adherence to use and the prevention of unintended pregnancies (1). High method satisfaction correlates to the user experience and perception, including ease of use, high effectiveness, few side effects, and ability to choose a method independent of cost (2). In recent studies, long-acting reversible contraceptive (LARC) methods – including hormonal intrauterine devices (IUDs), copper IUDs, and contraceptive implants – have been found to have higher continuation rates than oral contraceptive pills (3). Factors also associated with satisfaction and/or unintended pregnancies could include age, body mass index (BMI), educational level, and social background. Data from the contraceptive CHOICE project (4) showed that women of older reproductive age expressed higher satisfaction with all contraceptive methods compared with women of younger age groups. Satisfaction with LARC methods did not differ between age groups, but females aged 14–19 years were less likely to be satisfied with non-LARC methods when compared with women older than 25 years of age (5).

The global prevalence of obesity has nearly tripled in the last four decades (6). The World Health Organization defines obesity as a body mass index (BMI) > 30.0 kg/m^2^, and worldwide, over 300 million women are classified with obesity (7). The prevalence of overweight in Swedish fertile women ranged from 29% (16–29 years old) to 45% (30–44 years old) in 2021 (8). International studies have shown that body size does not affect women’s sexual behavior (9). However, women with a higher BMI have been reported to use less effective contraceptive methods and have higher rates of unintended pregnancies compared with women with a normal BMI (i.e. < 25.0 kg/m^2^) (10). A previous Swedish study reported that women with higher BMI were more likely to discontinue their contraceptive method within the first year (11), but the association between socioeconomic factors, including BMI, and choice, use, and satisfaction with contraception has not been assessed. In both the United States and western Europe, migrant women have higher rates of abortions compared with non-migrant women (12). Migrant women in Sweden, including adolescents, have been reported to be more likely to have induced abortion, compared with women born in Sweden (13).

An unmet need for contraception is defined as sexually active women who do not desire pregnancy and are not using any contraception. Among Swedish women, this unmet need has risen from 15.2% in 2017 (14) to 17.2% in 2021 (15), surpassing the average of 7% observed in other northern European countries (16). Given the increase of unmet need for contraception in Sweden (15), examining factors associated with contraceptive choice and contraceptive satisfaction is imperative.

In this cross-sectional study, we aim to explore how sociodemographic characteristics affect women’s use and level of satisfaction with different contraceptive methods.

## Materials and methods

### Study design

This cross-sectional study was conducted using data collected during a randomized controlled trial (RCT) in the region Örebro County, Sweden, with the aim to compare two different counseling models, standard counseling with counseling using the method Reproductive Life Plan (RPL) (17, 18).

Two months post-counseling, a follow-up questionnaire including questions regarding satisfaction with contraceptive methods was sent to the participants. Participants who completed the follow-up questionnaire are included in this cross-sectional study. The main RCT trial, including the full methodology, has been published elsewhere (17, 18).

### Study setting & sample

Örebro County, located in central Sweden, has a population of approximately 305,000 inhabitants. The county encompasses a mix of urban and rural municipalities. Örebro is the county largest city with a university and a tertiary university hospital, both of which play a central role in the region’s healthcare infrastructure.

**Figure 1:**
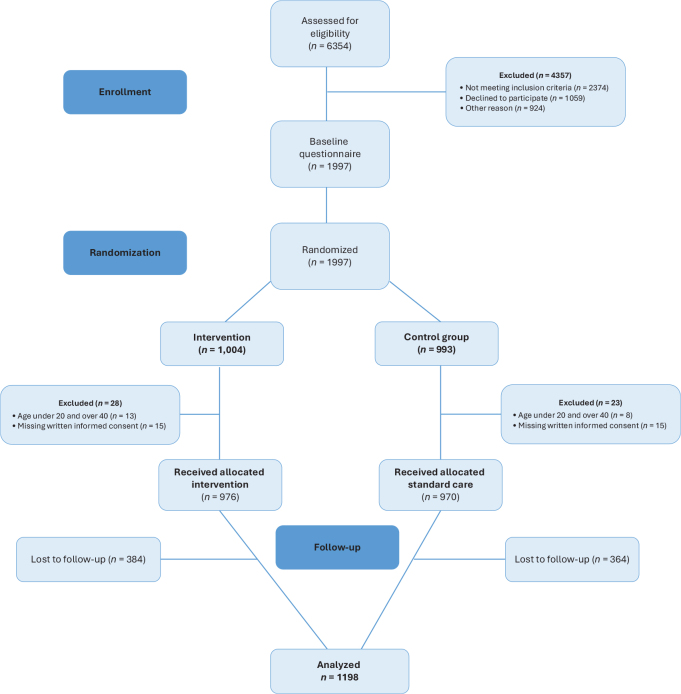
Flow chart for the inclusion of the study population.

Inclusion criteria were women aged between 20 and 40 years and having the ability to read and understand Swedish. Out of the participants enrolled in the original RCT trial, 1,198 (61.6%) responded to the follow-up questionnaire and, thus, included in the present study. This study was conducted between February 2015 and March 2016. The flow of participants is presented in Figure 1. Sociodemographic characteristics of the study population are displayed in Table 1.

**Table 1:** Background characteristics of study population.

Sociodemographic factor	Descriptive statistics	Estimate
**Age**	Median (IQR)	26 (9)
	Frequency: 20–26 years (%)	652 (54.4)
	26–40 years (%)	522 (43.6)
**BMI**	Median (IQR)	23.5 (4.9)
	Frequency: 15–24.99 (%)	789 (67.7)
	25–40 (%)	375 (32.3)
**Relationship status**	Frequency: In a relationship	927 (77. 4)
	Single	266 (22.2)
**Education**	Frequency: Up to high school	715 (59.7)
	Above high school	483 (40.3)
**Place of birth**	Frequency: Nordic Country	1135 (94.7)
	Non-Nordic Country	58 (4.8)
**Occupation** ^a^	Frequency: Employed	644 (53.8)
	Student	361 (30.2)
	Parental-leave	123 (10.3)
	Unemployed	32 (2.7)
	Sick leave	28 (2.2)
	Other	9 (0.8)

IQR: interquartile range.

a The question did not display a free text field.

As all questions were not mandatory, the total sums vary due to some participants not completing the question in the survey.

### Statistical analysis

To assess the main outcome of the current study and the level of satisfaction with ongoing contraception, the results were based on the question ‘To what extent are you satisfied with your contraceptive method?’. Participants could respond with one of the following options: ‘very satisfied’, ‘satisfied’, ‘neither satisfied nor dissatisfied (neutral)’, ‘dissatisfied’, or ‘very dissatisfied’. For analysis, the responses were dichotomized as ‘satisfied’ (including ‘very satisfied’, ‘satisfied’, and ‘neutral’) and ‘dissatisfied’ (including ‘dissatisfied’ and ‘very dissatisfied’). Furthermore, we dichotomized the sociodemographic variables ‘BMI’ (15–24.9 and 25–50), age (20–26 and 27–40 years), level of education (up to high school and above high school), and country of birth (Nordic country and non-Nordic country).

BMI was categorized at the threshold between normal weight and overweight, a widely accepted cutoff in both clinical and public health contexts. Age was divided at the median (26 years), aligning with Sweden’s contraceptive subsidy policy, which provides financial support up to that age. Initially, we explored a more detailed classification, dividing BMI into multiple categories and age into smaller groups to enable a finer-grained analysis. However, this approach resulted in excessively small subgroups, reducing statistical power and limiting the reliability of our findings.

We used descriptive statistics to present frequencies and proportions and chi-square tests to assess differences in sociodemographic characteristics, contraceptive choice, and satisfaction with ongoing contraceptive use. Logistic regression was used to calculate crude and adjusted odds ratios with 95% confidence intervals (CIs) to assess sociodemographic factors associated with contraceptive use and satisfaction, adjusting for BMI, education, age, country of birth, and the RLP intervention.

Statistical analyses were performed in SPSS Statistics, version 25 (IBM Corp., Armonk, NY, USA). A two-sided *P-*value < 0.05 was considered statistically significant.

## Results

The response rate for most questions was high, ranging from 95.5 to 100%. Compared with responders, the non-responders to the questionnaire were characterized by lower educational attainment, a higher prevalence of smoking and snuff (nicotine pouches) use, a greater likelihood of being employed, and a higher proportion of individuals born in a non-Nordic country. Additionally, non-responders were more likely to have been pregnant, given birth, and experienced abortion and miscarriage. Full non-response analysis is published in the main RCT trial (17, 18).

### Satisfaction and sociodemographic characteristics

**Figure 2:**
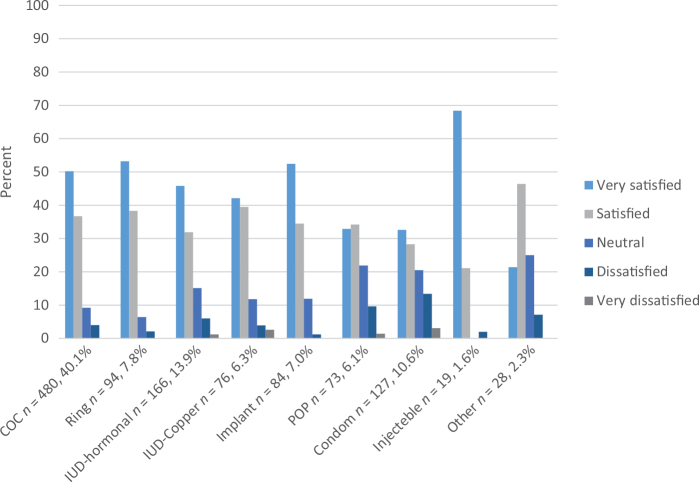
Satisfaction with ongoing contraceptive methods in study population. COC: combined oral contraceptives; IUD: intra uterine device; POP: progestin only pill; DMPA: depot medroxyprogesterone acetate.

Among participants who reported their satisfaction with their ongoing contraception, 81.3% (932/1,147) were either ‘very satisfied’ or ‘satisfied’. The distributions of satisfaction levels for the different contraceptive methods are presented in Figure 2.

When we analyzed levels of satisfaction and BMI, using all four BMI groups separately (underweight, normal weight, overweight, and obesity), the levels of satisfaction tended to get lower with an increasing BMI (*P* = 0.015) (Supplement 1), but when dichotomized (BMI ≤ 24.9 vs. BMI ≥ 25.0), there was no statistically significant difference in satisfaction.

There was no statistically significant difference in satisfaction when analyzing the demographic characteristic age in four age groups (Supplement 2). However, when dichotomized, being older, that is, aged 27–40 years, was associated with being slightly less satisfied with the ongoing contraception compared with being younger (91.5% vs. 94.8%, adjusted odds ratio [aOR] 0.55, 95% confidence interval [CI] 0.33–0.94) (Table 2). Educational level or being born outside of Nordic countries did not influence the level of satisfaction.

**Table 2:** Satisfaction with ongoing contraceptive use by sociodemographic and background characteristics.

Characteristic	Satisfied^a^	Dissatisfied^b^	Sum	OR (95% CI)	aOR^c^ (95% CI)
**Age**					
Age 20–26 (*n*, %)	601 (52.8)	33 (2.9)	634 (55.7)	Ref	Ref
Age 27–40 (*n*, %)	462 (40.6)	43 (3.7)	505 (44.3)	0.59* (0.37–0.94)	0.55* (0.33–0.94)
**BMI**					
BMI 15–24.9 (*n*, %)	720 (63.8)	48 (4.3)	768 (68.1)	Ref	Ref
BMI 25–50 (*n*, %)	335 (29.7)	25 (2.2)	360 (31.9)	0.89 (0.54–1.47)	0.92 (0.55–1.53)
**Education**					
Up to high school (*n*, %)	645 (55.5)	51 (4.4)	696 (59.9)	0.78 (0.48–1.26)	0.67 (0.39–1.15)
Above high school (*n*, %)	439 (37.8)	27 (2.3)	466 (40.1)	Ref	Ref
**Place of birth**					
Nordic Country (*n*, %)	1028 (88.9)	76 (6.6)	1104 (95.4)	Ref	Ref
Non-Nordic Country (*n*, %)	51 (4.4)	2 (2.6)	53 (4.6)	1.88 (0.45–7.89)	3.28 (0.45–24.22)

OR: odds ratio; aOR: adjusted odds ratio; CI: confidence interval.

* Statistically significant.

Data are *n* (%) if not stated elsewise.

a Includes the variables ‘very satisfied’, ‘satisfied’, and ‘neutral’.

b Includes the variables ‘dissatisfied’ and ‘very dissatisfied’.

c Adjusted odds ratios were obtained through a multivariate logistic regression model adjusted for BMI, educational level, age, country of birth, and the intervention.

### Contraceptive method use

The most common contraceptive method used at the latest sexual intercourse was combined oral contraception (COC), followed by the hormonal IUD and condom (Figure 2).

Participants with BMI ≥ 25.0 more often reported the use of LARC compared with participants with BMI ≤ 24.9 (33.9% vs. 25.2%; aOR 1.68, 95% CI 1.24–2.28) (Table 3). When analyzing participants with BMI ≥ 30 separately, 44.8% reported the use of LARC, 18% use of progestin only pill (POP), and 2.6% use of COC.

**Table 3:** LARC use and body mass index (BMI).

	Total *n*	LARC *n* (%)	No LARC n (%)	Crude OR (95% CI)	Adjusted OR (95% CI)
BMI 15–24.99	763	134 (17.6)	629 (82.4)	Ref	Ref
BMI 25–50	375	105 (28.0)	270 (72.0)	1.83 (1.36–2.45)	1.68 (1.24–2.28)*

OR: odds ratio; CI: confidence interval; BMI: body mass index; LARC: long-acting reversible contraception.

* Adjusted for age, educational level, country of birth, and intervention.

Fewer participants with BMI ≥ 25 compared with participants with BMI ≤ 24.9 used COC (aOR 0.6, 95% CI 0.46–0.78), while more used an IUD or implant (aOR 1.95, 95% CI 1.22–3.11). In addition, participants with BMI ≥ 25.0 to a higher extent than those with a BMI ≤ 24.9 reported use of no method at all (aOR 1.56, 95% CI 1.01–2.43), as did participants in the age group 27–40 compared with 20–26 years (aOR 1.63, 95% CI 1.01–2.61).

Participants in the age group 27–40 years more often reported the use of LARC (34.7% vs. 21.5%; aOR 1.92, 95% CI 1.44–2.56) compared with participants in the younger age group. Reportedly, the use of IUDs was more common in the older age group, while the participants in the younger age group were more likely to report the use of implants or COCs (Table 4).

**Table 4:** Use of different contraceptive methods by sociodemographic and background characteristics.

Method	Age			BMI			Education			Place of birth		
	20–26 *n* = 643	27–40 *n* = 518	*P	15–24.9 *n* = 778	25–50 *n* = 372	*P	Above high school *n* = 707	Up to high school *n* = 477	*P	Nordic *n* = 1122	Non-Nordic *n* = 57	*P
COC*	306 (47.7)	165 (31.8)	*< 0.001*	349 (44.9)	116 (31.2)	*< 0.001*	298 (42.2)	182 (38.2)	*0.168*	460 (41.0)	19 (33.3)	*0.273*
Vaginal ring	67 (10.4)	27 (5.2)	*0.002*	72 (9.3)	20 (5.4)	*0.015*	61 (8.6)	33 (6.9)	*0.325*	90 (8.1)	3 (5.3)	*0.617*
POP	37 (5.7)	35 (6.7)	*0.466*	40 (5.1)	30 (8.1)	*0.064*	44 (6.2)	29 (6.1)	*1.000*	72 (6.4)	0 (0.0)	*0.044*
Injection (DMPA)	4 (0.6)	15 (2.9)	*0.004*	8 (1.0)	10 (2.6)	*0.042*	11 (1.5)	8 (1.7)	*1.000*	18 (1.6)	1 (1.8)	*0.615*
Hormonal-IUD	57 (8.9)	106 (20.6)	*< 0.001*	104 (13.4)	61 (16.4)	*0.177*	79 (11.3)	87 (18.2)	*< 0.001*	160 (14.1)	4 (7.0)	*0.168*
Implant	58 (9.0)	25 (4.8)	*0.008*	45 (5.8)	37 (9.9)	*0.014*	61 (8.6)	23 (4.8)	*0.015*	73 (6.5)	11 (19.3)	*0.002*
Copper-IUD	25 (3.9)	50 (9.7)	*< 0.001*	47 (6.0)	28 (7.5)	*0.371*	41 (5.8)	35 (7.3)	*0.334*	73 (6.5)	3 (5.3)	*1.000*
Condom only	36 (5.6)	29 (5.6)	*0.005*	42 (5.4)	23 (6.2)	*0.080*	49 (6.9)	21 (4.4)	*0.013*	64 (5.7)	6 (10.4)	*0.386*
Other	14 (2.2)	12 (2.3)	*1.000*	17 (2.2)	8 (2.2)	*1.000*	16 (2.3)	11 (2.3)	*1.000*	25 (2.2)	2 (3.5)	*0.643*
No method	39 (6.0)	54 (10.4)	*0.004*	54 (6.9)	39 (10.5)	*0.043*	47 (6.6)	48 (10.1)	*0.020*	87 (7.9)	8 (14.1)	*0.144*

BMI: body mass index; COC: combined oral contraceptives; IUD: intra uterine device; POP: progestin only pill; DMPA: depot medroxyprogesterone acetate.

* Data are *n* (%) if not stated elsewise.

Note: Fisher´s Exact Test was used to determine if there was a significant association between sociodemographic variables and contraceptive method used.

As all questions were not mandatory, the total represents the number of participants completing the question in the survey.

Participants from non-Nordic countries more often reported the use of implants compared with participants from Nordic countries (*P* = 0.002), and no woman born outside the Nordic countries reported the use of POP (*P* = 0.044) (Table 4).

## Discussion

In this cross-sectional analysis, the most common contraceptive method used at last intercourse was the COC, followed by LARCs with significant differences in the utilization observed across different demographic factors. The use of LARC was more common among women with higher BMI and higher age. Overall, satisfaction with contraceptive methods was high; however, women aged 27–40 reported lower satisfaction with their current method compared with younger women. When we grouped into two classes, satisfied or dissatisfied, participants who stated that they were neither satisfied nor dissatisfied (neutral) with their contraceptive method were classed as satisfied. The rationale for this decision was that dissatisfaction is related to discontinuation of contraceptive use (1), and that persons who are neutral most probably will not discontinue the use because of dissatisfaction. BMI, educational level, and place of birth did not have a significant impact on the satisfaction of method.

When BMI was analyzed in four groups, a trend emerged, indicating a decrease in satisfaction as BMI increased. More specifically, individuals classified as overweight or obese reported lower satisfaction levels than those in the normal weight or underweight categories. This pattern suggests a potential link between higher BMI and reduced overall satisfaction. However, due to the small groups, the results must be interpreted with caution, especially women in the fertile age normally display an increasing BMI with increasing age (19).

Even though the level of satisfaction was generally high, older participants displayed a lower level of satisfaction with their use of ongoing contraceptive method compared with participants of younger age. This finding was unexpected, as prior studies have reported higher satisfaction among older women (5, 20). However, this finding might reflect the Swedish contraceptive eligibility guidelines issued in 2014 (21), which, for the first time, emphasized the use of IUDs also in nulliparous women and women in the younger age groups (2, 22). These recommendations have led to an increase in IUD uptake among young women in Sweden, especially hormonal-IUDs, and these methods have shown to have the highest method satisfaction and continued use (3).

Participants with a BMI ≥ 25 more often reported the use of LARC, which is in accordance with results from previous studies (10, 23). This could be explained by the current Swedish and European guidelines discouraging the use of combined hormonal contraceptives to women with obesity. The European medical eligibility criteria on combined hormonal methods state that BMI > 30 is ‘a condition for which the theoretical or proven risks usually outweigh the advantages of using the method’ due to the increased risk of venous thromboembolism associated with the use of combined hormonal contraceptives in obese women (10, 23, 24). If access to LARC or POP methods is not highly available or the overweight women find these methods ineligible, the guidelines consequently leave them with fewer contraceptive options (21). However, the Swedish guidelines in general emphasize the use of LARC as a first-line option independent of age, reproductive history, or bodyweight, which could be reflected in our results.

Few participants with BMI ≥ 25 used POP. A previous Swedish study showed that POP was the most commonly used contraceptive method by women with overweight or obesity. It was also suggested that women with higher BMI were more likely to discontinue their contraceptive method within the first year due to bleeding disturbances (11). When we analyzed participants with a BMI ≥ 30 separately, the use of POP was increased compared with participants with lower BMI.

The group of participants with overweight or obesity reported the higher use of LARCs, which are known to be methods with high user satisfaction (2, 22). On the other hand, this group, despite not having any pregnancy intentions, was at greater odds of reporting non-use of contraception compared with normal weight women. This finding is reflected in other studies, which suggest that women with obesity more often rely on less effective methods such as withdrawal or do not use any contraceptive methods at all (10, 23).

Women born outside the Nordic countries showed a slightly different pattern in the use of progestogen only methods. More women used an implant, and no women reported the use of POP. This is an interesting and gratifying finding. A previous, however old, study has reported higher abortion rates in immigrant women in Sweden (25), and it is most desirable that women in high-risk groups for unintended pregnancies use high effective contraception, that is, LARC. The results must, however, be interpreted with caution due to small groups.

Identifying individuals at higher risk for unintended pregnancy and understanding contraceptive use patterns are key to effective public health interventions. Tailored education can enhance acceptance, particularly among diverse age groups and minority populations. Since satisfaction influences continued use, a person-centered approach helps providers address women’s needs more effectively.

Perceptions of ‘satisfaction’ vary widely, shaped by personal experiences often unknown to providers. To improve contraceptive use and reduce unintended pregnancies, further research is needed on cultural and sociodemographic factors affecting contraceptive choices.

### Strengths and limitations

A key strength is that this study is based on data from a randomized controlled study in which all maternal clinics in a certain health care region participated. All women seeking contraceptive counseling during the study period were screened for eligibility and invited to participate, resulting in a representative study population in terms of different social backgrounds, educational levels, and occupations. The response rate of the questionnaire was 61.6%, which could be considered satisfactory, as it has become more difficult to recruit people to survey studies. In Sweden, the response rate in public health surveys decreased from 60.8% in 2004 to 47% in 2016 (26). However, the fact that responders were more likely to have higher education levels, lower rates of smoking and snuff use, more likely to be students, and be born in a Nordic country (17) indicates that the material might differ from the general population and possibly affect the results. On the other hand, clinical trial participation remains largely inaccessible to historically underrepresented groups, including those with lower income and education (27).

In the main trial, the intervention relied on direct communication between the midwife and client, and only Swedish-speaking women were eligible for inclusion. This limited the number of non-Nordic participants, making it difficult to draw conclusions regarding ‘country of birth’. Additionally, this study only included women aged 20–40. These inclusion criteria contribute to selection bias, potentially resulting in the generally high satisfaction levels. The exclusion of adolescents, who might have different contraceptive preferences and needs, may impact the generalizability of findings.

Finally, as a cross-sectional study, our data represent a single point in time and may not fully capture evolving societal trends, healthcare practices, or contraceptive preferences. This limits the study’s ability to reflect current conditions or predict future patterns.

## Conclusion

In this cross-sectional study, being of older age and having a higher BMI were associating demographic factors to higher use of LARC.

No connection was seen between BMI, level of education, or place of birth and contraceptive method satisfaction; however, women aged 27–40 years reported lower satisfaction. Previous studies are drawing on higher satisfaction among users of LARC methods and older women within the reproductive life span. Thus, our contrasting findings underscore the complex interplay of sociodemographic influences on contraception experiences. Future studies could include different demographic factors and take them into consideration when reporting on user satisfaction and continued use, and when designing new interventions to target individual needs in contraceptive counseling and prescription.

## Data Availability

The data are not available to be shared without obtaining ethical approval for changes in the data sharing process.
